# Healthcare transformation journey in the Eastern Region of Saudi Arabia: an overview, challenges and lessons learned

**DOI:** 10.25122/jml-2023-0010

**Published:** 2023-04

**Authors:** Lamees Yousef, Dannah AlAngari, Rahaf AlShehri, Bader AlSharif, Omar Bayameen, Zeinab Alnemer

**Affiliations:** 1 Model of Care Hub Department, Health Holding Company, Khobar, Saudi Arabia; 2 College of Public Health, Imam Abdulrahman Bin Faisal University, Dammam, Saudi Arabia

**Keywords:** Saudi Arabia, health care, Model of Care (MoC), Vision Realization Office (VRO), System of Care (SoC), global health, health policy, delivery of healthcare, AHC – AlAhsa Health Cluster, EHC – Eastern Health Cluster, HBHC – HaferAlbatin Health Cluster, HIS – Health Information Systems, ICCPs – The Integrated Clinical Care Pathways, KPIs – Key Performance Indicators, MDT – Multidisciplinary Design Team, MoC – Model of Care, MoH – Ministry of Health, NCDs – non-communicable diseases, OSC – One Stop Clinic, PHCs – Primary Healthcare Centers, PMO – Project Management Office, SoC – System of Care, VRO – Vision Realization Office, WHO – World Health Organization

## Abstract

The Kingdom of Saudi Arabia has embarked on a transformation journey referred to as "Vision 2030", which commenced in June 2016. The healthcare sector is currently going through a radical transformation under this Vision. The new Model of Care shifts the focus of the healthcare sector towards proactive care and wellness, aiming to achieve better health, better care, and better value. This paper aims to provide an overview of the Model of Care and review its achievements and progress in the Eastern Region. The paper will further discuss the challenges faced and lessons learned through the implementation process. Internal documents were reviewed, and a comprehensive literature search was undertaken in relevant search engines and databases. Some of the successes of the Model of Care implementation include improved data management, collection and visualization, and better patient and community engagement. Nevertheless, there is a sense of urgency to face the many challenges identified in the Saudi Arabian health system over the coming decade. Although the Model of Care focuses on addressing these identified challenges and gaps, there are many difficulties facing its implementation in the country and several lessons learned during the first few years since its launch, which this paper mentions. Hence, there is a need to measure the successes of pathways and the overall impact of the Model of Care on both the healthcare provision as well as improved population health.

## INTRODUCTION

The progress made by the Kingdom of Saudi Arabia in healthcare over the last few decades has been nothing short of remarkable [1]. The healthcare system in the Kingdom has changed dramatically over the years, from being purely curative to reducing the effects of infectious diseases to, finally, focusing on preventative medicine [1]. Prior to 1925, the healthcare infrastructure was underdeveloped, and the resources were scarce, with only three hospitals in Mecca City [2]. The infrastructure improved in 1926 when the Kingdom established the Health Department under a royal decree [2,3]. The emergence of the Health Department marked the beginning of an established healthcare system in Saudi Arabia [4]. The Department was tasked with supervising the services provided by new hospitals and clinics in urban areas. To further raise the standard of healthcare, the Kingdom established a health council. The true turning point, however, was the establishment of the Ministry of Health (MOH) in 1950 under royal decree. Under this decree, the Health Department was responsible for providing health services to all citizens, which is still in effect today, and overseeing the care provided in public and private healthcare facilities [3]. The MoH also played a key role in the strategic planning, monitoring, and control of health services [5,6]. Healthcare is provided under the guidance of the MoH through a network of primary healthcare centers (PHCs) and hospitals. Other governmental agencies that provide healthcare services to their own staff include the Ministry of Education, the Ministry of Defence and Aviation and the Ministry of Interior, and the Red Crescent. In 1970, Saudi Arabia issued its first-ever five-year development plan to ensure progress in several areas, including healthcare [5]. Since then, healthcare has been prioritized in subsequent development plans, and the Kingdom witnessed a major shift in healthcare delivery, especially with the establishment of the required infrastructure for hospitals, PHCs, and research facilities [5,7]. The most recent data indicates that Saudi Arabia has a total of 498 hospitals with approximately 77 thousand bed capacity [8]. In addition, the healthcare system of the Kingdom was ranked by the World Health Organization (WHO) as number 26 among 191 countries [2]. In a relatively short period of time, the Kingdom has achieved remarkable accomplishments in healthcare worthy of recognition. Despite that, as any growing nation, the Kingdom faces inevitable challenges that have prompted a radical transformation in line with Vision 2030 goals.

### The reasons for change

There is a sense of urgency to face the many challenges identified in the Saudi Arabian health system over the coming decade [9,10]. As stated by Rahman&Qattan [7], the expectation of better quality care, the rising cost of healthcare, the general population growth, and the emerging lifestyle diseases are driving healthcare reform [7]. As of 2020, the estimated population of Saudi Arabia was 35 million people, with 33% below the age of 20 and less than 4% above the age of 65. However, by 2050, in addition to overall population growth, it is estimated that those over 65 will eventually make up 20% of the population. The increase in the older adult population is expected to lead to an increase in non-communicable diseases (NCDs), driving up the cost of healthcare [11].

Furthermore, Saudi Arabia faces significant challenges due to the prevalence of sedentary lifestyles, with 75% of the population receiving no regular check-ups and 60% being overweight or obese [11]. Therefore, the most substantial burden is the ever-increasing rate of chronic conditions in the Saudi population, which is projected to rise further by 2030. In addition, the number of premature deaths attributed to chronic conditions is almost double that of developed countries [11]. Primary healthcare in the Kingdom remains inconsistent and inadequate to meet the population's needs, with underserved secondary and tertiary hospitals and associated resources [12,13]. Thus, interventions are needed to reduce the cost of treating diseases and prevent non-communicable diseases, as the healthcare system must respond to the changing population demographics and disease patterns [4].

### The transformation journey

The Kingdom of Saudi Arabia embarked on a national transformation journey in June 2016 known as “Vision 2030.” The Vision focuses on three main objectives, creating a vibrant society, a thriving economy, and an ambitious nation [14]. To achieve this, the Kingdom launched transformation programs centered around eight main themes. These include transforming healthcare, improving living standards and safety, ensuring the sustainability of vital resources, social empowerment and non-profit sector development, achieving governmental operational excellence, improving labor market accessibility and attractiveness, enabling the private sector, and developing the tourism and national heritage sectors [10]. Within the Health Sector Transformation Program (HSTP), there are four key goals identified to drive the transformation of the health system to support the objectives of the Vision. These goals are to: improve the quality and efficiency of health services, promote the prevention of health risks, facilitate access to health services, and enhance traffic safety [10].

Several programs have been launched in line with the goals of Vision 2030. These programs were informed and directed by a redefined Model of Care (MoC) that shifts the overall focus of the healthcare system toward proactive care and wellness and aims to achieve better health, care, and value. It was designed around six systems of care to address the needs of all population segments [12]. It drives a shift in mindset, emphasizing integration and wellness through people activation and empowering citizens to play an active role in maintaining and improving their health. In addition, one of the main milestones that would be achieved through implementing MoC is enabling the clusters to provide healthcare services built upon value. This would be completed by linking the payment mechanism to the implemented clinical pathways and desired patient outcomes. Currently, Value in Health is guiding the national effort to achieve this future goal. Value in Health is an independent national center focused on improving value in health through policy development, knowledge management, and building capabilities that influence policy [15].

This paper aims to describe the MoC approach, methodology, implementation cycle, objectives and aims. It also identifies the MoC deliverables, challenges, and lessons learned within the Eastern Region of Saudi Arabia and ways to move forward.

## Material and Methods

A directed content analysis approach was applied to internal documents used within the MoC teams at the Clusters in the Eastern Region of Saudi Arabia. The goal of the directed approach was to describe the implementation of the MoC, the challenges faced, and the lessons learned. The key concepts were first agreed upon, then all relevant documents were reviewed, categorized, and analyzed according to the predetermined key concepts. In addition, data relating to the MoC, the healthcare transformation in Saudi Arabia, and the “Vision 2030” was collected from the Vision Realization Office (VRO) and the Ministry of Health (MoH). Different search engines and databases were used, such as Google Scholar, PubMed, CORE, Science.gov, and ScienceDirect. All collected data were analyzed and synthesized, and findings were reported.

## Results

### Challenges faced by the Saudi health system

The healthcare transformation in the Kingdom of Saudi Arabia in line with ‘Vision 2030’ was prompted by multiple concurrent factors concerning the main health indicators of the population and other health challenges, as mentioned earlier [12]. A national current state assessment revealed several significant challenges faced by the Saudi healthcare system [12], including the expected population growth by 2030, which involves a growing aging population, and the presence of foreign residents who make up 36.4% of the population, adding an extra burden to the healthcare system [16]. Additionally, Saudi Arabia provides free healthcare to visitors and pilgrims through MoH facilities [17]. In some years, foreign visitors can reach up to five million people, especially during high peak seasons or major religious events, such as Hajj and Umrah, putting extra pressure on the already strained healthcare system [18].

Furthermore, Saudi Arabia has a high prevalence of injuries and NCDs compared to international and regional standards [19]. Specifically, the Kingdom’s high prevalence of heart disease, stroke, diabetes mellitus, mental health, road traffic accidents, and congenital diseases increases the population of those living with one or more NCDs [19]. NCDs are considered a threat to the 2030 Vision, which aims to increase life expectancy from 75 to 80 years and reduce premature deaths from NCDs by one-third by 2030 [11].

Improving access to healthcare services requires equity in the distribution of healthcare facilities between and within regions across the country and equal access to health professionals, a current challenge in the Kingdom [11]. A study in Riyadh, Saudi Arabia, sheds light on factors affecting access to and utilization of PHCs in urban and rural areas [20]. The study found that urban respondents would like increased opening hours, particularly in the evenings. Factors affecting the rural population included the distance to the PHC center, sanitation, and the quality of services, including disease prevention and health promotion.

The healthcare sector faces an increasing expenditure, leading to a budget deficit due to an imbalance between revenue and expenditure. This has led to plans for the privatization of health services and facilities promoted by the government [21]. While oil is the backbone of the Kingdom's economy, relying solely on oil resources as the major contributor to financing the public health sector is unsustainable [1]. Thus, other sources of funding are necessary [21].

The challenges faced by the Saudi healthcare system, as outlined above, indicate that the current model is not sustainable, highlighting the need for a significant transformation of the health sector as a whole. This transformation is necessary to enhance sustainability, quality of care, access to care, and system efficiency [12].

### Model of care strategy and design

In order to work towards developing the strategy for implementing the Health Transformation, the VRO divided the process into seven themes: the new MoC, provider reforms, financing reforms, governance growth, private and third sector engagement, workforce development, and eHealth development [12]. The MoC focuses on enhancing personal value at an individual level by improving treatment and care methodologies. It aims to address challenges and gaps within the current healthcare system. The priority is to address challenges faced by all clinical pathways that affect the provision of care, including medication shortages, unclear clinical guidelines, inadequate patient flow and referral protocol, lack of patient understanding and knowledge, insufficient continuity of care and preventative services, lack of coordination between ministry hospitals and non-ministry organizations and, finally, lack of clear communication between providers and patients [12].

Since these concerns are shared among all clinical experiences within the health system, the goal of the transformation and the MoC program, in general, is to answer the following six questions [12]:

1. How will the system help to keep me well?
2. How will the system support me when I have an urgent problem?
3. How will the system support me to have a positive outcome from my planned procedure?
4. How will the system support me in safely delivering a healthy baby?
5. How will the system support me with my chronic conditions?
6. How will the system support me with compassionate care during the last phase of my life?

To improve the MoC design, data collection and assessment of the current state of the healthcare system were necessary. To determine indicators of the current state of healthcare, a survey was distributed within the Kingdom among over 60,000 citizens. In addition, over 2,500 healthcare professionals from all over the world participated in discussions, and over 1,000 were surveyed to understand the perspective of these target groups. The main objective was to identify possible solutions to the six main questions, which inspired the groundwork for the new MoC. The answers to these questions would eventually become the six Systems of Care. After laying the foundations for the discussion, and for the next six months after that, the MOH and the VRO led three national workshops to design a comprehensive health system for the Kingdom. The workshops comprised key stakeholders, including over 2,450 healthcare professionals representing different health system components from different regions of the Kingdom. The first phase of these discussions was meant to agree on and discuss the findings regarding the issues and concerns identified during the initial state assessment. The second workshop aimed to complete a list of interventions to address those concerns, which led to the development of the Systems of Care. The final workshop consisted of representation from each of the identified leads for each system, as well as international experts and other relevant stakeholders. It was meant to develop a clear and comprehensive MoC design, as well as how that design would interact with and support the health goals within the different contexts in the Kingdom. The MoC was officially launched by the MoH on the 23rd of April, 2017 [12].

### Final Model of Care design: systems and interventions

Finally, after the current state assessments, workshops, and discussions were completed, the new MoC in the Kingdom was designed based on 5 main principles [22]:

1. Empowering people to become active in their own and their family’s health;
2. Keeping patients informed of their health, treatment plans, medications, etc;
3. Creating a smooth and integrated patient journey within the health system;
4. Focusing on preventive care and supporting the health of the population as a whole;
5. Focusing on the patient during treatment and creating an outcome-based approach.

**Figure 1:**
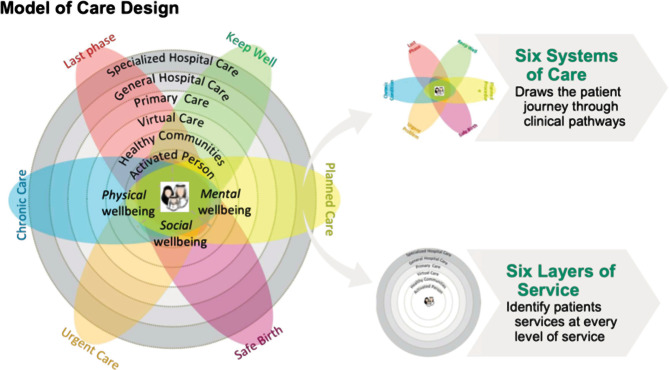
Model of Care and Systems of Care [12].

Ultimately, the MoC was designed with the patient at the forefront of the discussion. The final design comprised six Systems of Care over multiple ‘layers’ (Figure 1): Keep Well, Planned Care, Safe Birth, Chronic Care, Urgent Care, and Last Phase [12]. Within these six systems, there were 42 identified interventions, 27 grouped between the systems, and 15 considered cross-cutting across 2 or more systems. An intervention was defined as a strategy or program intended to address certain issues or concerns. The purpose of the intervention is to prompt a change in patient outcomes in response to either behavioral or clinical changes to a patient pathway or to reduce cost within that ‘System’ [22].

### Systems of Care

Each System of Care (SoC) has a purpose and objective for the population. Within each system, specific interventions are designed to address concerns within the field of the respective system [23]. The systems, their descriptions and purpose, and their ground interventions are outlined in Table 1, which also shows how they are categorized under different Systems of Care. The table also lists cross-cutting interventions that apply to one or more systems [12].

**Table 1:** Systems of Care and interventions

System name	Description	Intervention
**Keep Well**	This system is designed to support a healthy community and create an environment for people to stay well and get well again after injury or sickness. The purpose is to educate patients and empower them to utilize the resources of the community.	1. Health coach programs;
		2. Community-based wellness programs;
		3. Workplace wellness programs;
		4. School wellness programs;
		5. Healthy food promotion;
		6. Health edutainment programs;
		7. Promoting the Saudi Centre for Disease Control.
**Planned Care**	This system is designed to support patients who have received planned procedures to ensure that the patient has a positive outcome and gets the necessary high-quality care before, during, and after the procedure.	8. One-stop clinics;
		9. Pathway optimization;
		10. Length of stay reduction initiatives;
		11. Step-down and post-discharge services.
**Safe Birth**	This system is designed to support women of child-bearing age to have a safe experience before marriage, before conception, and during and after pregnancy to deliver healthy infants and have a positive experience.	12. Premarital screening;
		13. Preconception care services;
		14. Maternity care services;
		15. National birth registry;
		16. Postnatal care services;
		17. Well baby clinics;
		18. Neonatal care services.
**Urgent Care**	This system is focused on addressing the processes and resources of patients with urgent medical issues and ensuring that they receive timely and appropriate care. In addition, it ensures that patients receive the necessary resources and support in their community.	19. A resource control center;
		20. Urgent care clinics;
		21. Population-based critical care centers.
**Chronic Care**	This system is designed to support patients with chronic conditions. Its objective is to provide integrated care across all levels necessary for screening, preventing, and treating chronic diseases. The system strives to empower patients to understand, care and advocate on their behalf.	22. Chronic disease screening;
		23. Case coordination;
		24. Continuing care services.
**Last Phase**	This system is designed to support patients and their loved ones in the last phase of their lives. It works to provide and empower patients to make their health decisions in the final days of their lives and support them to have a more comfortable experience.	25. Patient and family support;
		26. Hospice care services;
		27. Multidisciplinary team development.
**Cross-Cutting Interventions**	These are the interventions integrated within at least 2 systems of care to support their implementation.	28. Health in all policies;
		29. Virtual self-care tools;
		30. Virtual education and navigation tools;
		31. Health hotline services;
		32. Healthy living campaigns;
		33. School education programs;
		34. Enhanced primary care services;
		35. Enhanced home care services;
		36. Resource optimization;
		37. Integrated personal health records;
		38. National referral networks;
		39. National guidelines;
		40. Outcomes monitoring;
		41. Systematic data collection;
		42. Health research programs.

### Health clusters and first pilot projects

As part of the health transformation journey, the MoH segmented healthcare facilities and PHCs into groups based on geographical regions and under a single administration. The groups were launched into what is now known as Health Clusters [24]. The aim of this concept is to promote integration in healthcare provision and allow patients to seek medical care within the health facility networks. To become a formal cluster, the MoH has established a set of formal leadership assignments, processes, and structures that must be in place to be considered officially launched. However, some clusters could begin MoC implementation before officially being declared a cluster to expedite the transformational objectives. MoC planning and implementation are independent of cluster status.

The MoC pilot projects were implemented in the clusters/un-clustered areas by first nominating anMoC steering committee, which is accountable for the MoC implementation in the designated cluster/un-clustered area. Its main objective is to manage and monitor the implementation of the MoC pilots and allocate the necessary resources for the workforce and equipment. The steering committee is also the approving body for the MoC 2-year strategic plan as well as the MoC pathways.

### Model of Care implementation

The final MoC program provides a broad and generic guide for each intervention, specifying how to utilize these interventions to serve the health needs of any community through clinical pathways. All MoC clinical pathways and initiatives are implemented in phases following the process defined in the MoC implementation cycle.

### Model of Care clinical pathways

The clinical pathways methodology was adopted to enhance and operationalize the MoC initiatives and care delivery. Clinical pathways describe the healthcare journey for each population segment based on their health needs and necessary services required at different levels of care to achieve optimal clinical outcomes. As an extension of the MoC program, a detailed and standardized approach was implemented to reduce variability in clinical care and achieve efficient, timely, and patient-centered healthcare.

To design a clinical pathway, the clusters must follow the Pathway Development Framework outlined in the Pathway Design Manual issued by the national MoC design team. According to the Pathway Design Manual, the first step in designing a clinical pathway is assigning a Multidisciplinary Design Team (MDT) from the clusters to design the pathway. The MDT is also responsible for regularly revisiting the pathways for continual improvement throughout the implementation cycle. For the specified health condition/need, the MDT works on collecting available data and selecting appropriate clinical guidelines. Next, the MDT looks into previously developed pathways from either national or international practices to inform comprehensive pathway development. The MDT then maps out the current clinical pathway within the cluster and determines the areas for improvement. Finally, the MDT designs the future pathway to cover the gaps in care by including specific SoC interventions and enhancing full integration between service layers.

### Clinical pathways and Integrated Clinical Care Pathways (ICCPs)

The Integrated Clinical Care Pathways (ICCPs) approach is an improvement on the previous methodology of clinical pathways, which ensures stronger alignment and standardization at the national level. ICCPs were developed to enable the measurement of clinical outcomes, efficiency, cost, and workforce. An ICCP is a written document designed by several key stakeholders, including the patient, at the national level to describe a holistic patient's journey for a specific disease from the beginning of their ailment through diagnosis, treatment, and rehabilitation or resolution. One of the special elements added to the ICCP approach is standardized Key Performance Indicators (KPIs). Each ICCP includes multiple process and outcome KPIs that should be regularly measured and reported at the national levels after the kick-off of the implementation. ICCPs are meant to be clear, easy to understand, and flexible to give healthcare providers the chance to customize care pathways based on the specific needs of their population and the capacity of healthcare professionals within their clusters.

### 2-year strategic plan

Once a cluster/un-clustered area is ready to plan the implementation of the MoC, they begin by developing a two-year strategic plan referred to as the “Rollout Plan.” The MoC Rollout Plan aims to use the 42 MoC initiatives to meet the cluster’s population needs. As a result, each MoC Rollout Plan is unique to each cluster.

The Rollout Plan follows a standardized five-step process. The first step is setting the stage, where the MoC’s cluster lead assigns one lead per SoC. Each lead is responsible for one of the six systems and nominates 10-13 task force members of different specialties and layers of care. Once the team is finalized, the SoC lead conducts the MoC onboarding for their respective teams.

The second step of the Rollout Plan involves conducting a gap analysis, which consists of at least six workshops for each SoC. The workshops are conducted using a standardized framework that supports the team to reflect and identify the local gaps and needs of each initiative for every SoC. The comprehensive framework covers service portfolios, processes, systems, governance, people, skills, equipment, data, pharmaceuticals, infrastructure, and facility supplies. It also covers gaps and regulations related to patient engagement. Once the workshops are completed, the outcomes are sent to the task force for initial approval, then to the SoC leads for sign-off, and finally to the MoC lead. The task force then develops a gap summary report.

The third step involves the development of the MoC blueprints. This process also starts with at least six workshops, one for each SoC. During these workshops, the team begins by reviewing the gap assessment to understand the MoC interventions. The team members then design complete innovative pathways for the patient’s journey based on the need of the cluster. The blueprints also demonstrate the key challenges per SoC and the goals for the next two years. The blueprints describe the number of pathways, what initiatives to implement, and the number of facilities to implement the pathway or project.

The last two steps occur after the approval of the blueprints. The MDT is then responsible for developing a project charter for every project listed within each SoC blueprint. These project charters contain the scope of work, strategic objectives, KPI deliverables list, risks and mitigation, and benefits to be realized from the project. It also defines the project timeline, milestone list, and high-level resource analysis to identify the pathway needs for workforce and equipment. Once all steps are finalized, the team prioritizes the pathways based on their impact and ease of implementation. Finally, an integration matrix is completed to estimate how the pathways will interact with each other during the design and implementation phases.

Following the approval of the Rollout Plan for a given cluster, the next step is to assign clear roles and responsibilities for a Project Management Office (PMO) sponsored by the MoC. The PMO setup and support plan clarifies how the MoC team should operate on a day-to-day basis with respect to the MoC and cluster needs. This plan contains the team structure, job description, assignment of team members, reporting line, escalation, delegation process, and more. In addition to the governance and set up, the plan will clarify the level of support needed to ensure a successful implementation of the MoC projects and the priority of each pathway. The PMO setup and support plan should generally be detailed enough to cover the entire MoC project cycle, from planning to designing, piloting, scale-up, and rollout.

### Implementation cycle

The implementation of MoC pathways and initiatives follows a defined cycle that starts from the design phase and continues until they are integrated as business as usual and continuously improved. There are four main phases in any pathway implementation cycle: Preparation, Scale-up, Rollout, and Handover (project closure). Each phase includes certain activities that need to be completed to move to the next phase.

#### Preparation phase

There are two main activities within the preparation phase: Design and Pilot. In the Design phase, the MoC team will identify the design team to conduct the pathway design workshops and complete all the elements within the pathway design according to the adopted pathway development framework. Any finalized pathway design will be approved at the MoC team and the cluster level.

The team will work on conducting a current state assessment of the designated facilities tasked with implementing the pathway. This activity will provide the team with a better understanding of the readiness of the facilities and the possible resources needed to support the implementation.

To prepare for the pilot of the pathway, the team will develop an implementation plan detailing all the needed activities and resources to initiate the implementation kick-off. Completing all the activities in the implementation plan ensures the readiness to begin piloting the pathway.

The aim of piloting pathways is to test the implementation on a small scale initially and ensure a gradual but consistent expansion of the implementation across the facilities. Piloting pathways gives the MoC team in-depth insight into the task force capacity needed, how to optimize resource usage, and the impact of a facility’s infrastructure on the overall implementation. The pilot is considered officially commenced once patients are enrolled in the pathway.

#### Scale-up phase

The Scale-up phase involves expanding the implementation of the pathway to cover 70% of the targeted facilities. During this phase, the MoC team conducts improvement sessions to assess the current implementation and identify areas for improvement. Any necessary improvements are then incorporated into the updated pathway design and implementation plan. The scaling-up process is gradual across the targeted facilities over a specific period.

#### Rollout phase

Rolling out the implementation of the pathway means expanding the scope of the implementation to cover 90% of the targeted facilities. The MoC team is expected to conduct another improvement session to assess the current implementation and identify areas for improvement.

#### Handover phase

Finally, when all the objectives of the pathway implementation are achieved and the implementation covers 100% of the targeted facilities, project closure is triggered, and the handover phase is initiated. Project closure will ensure the readiness of the pathway to be moved to ‘business as usual’ based on the pathway success criteria. Completing all the activities within the project closure will initiate handover process planning in which the MoC team, along with any other stakeholders, will work on ensuring a successful transition of the project activities within the pathway to the respective operational arms within the health cluster. The completion of these project activities will enable the MoC team to focus on identifying new transformational pathways that need to be designed according to the gaps and health needs of the community, while the maintenance and operation of the pathway will be managed by the cluster after finalizing the handover process.

### Eastern Region progress

The MoC transformation came as a mandate from the VRO in 2017 [12]. For care delivery, regions in Saudi Arabia were sectioned into clusters defined by geographical boundaries, which included multiple cities and rural communities. Each cluster comprises hospitals, PHCs, schools, home care, medical facilities, and medical cities and is governed by a C-suite and board [25]. The ultimate goal of the cluster model is to provide health services to a defined population within the cluster catchment areas [24]. The Eastern Province is the largest geographical region in Saudi Arabia, which represents 26% of the Kingdom [26]. According to the General Authority for Statistics, the Eastern Province had a population of 4,105,780 as of 2017 [16]. The most populous cities are Al-Hasa and Dammam, with populations of 1,041,863 and 1,024,409, respectively.

**Figure 2:**
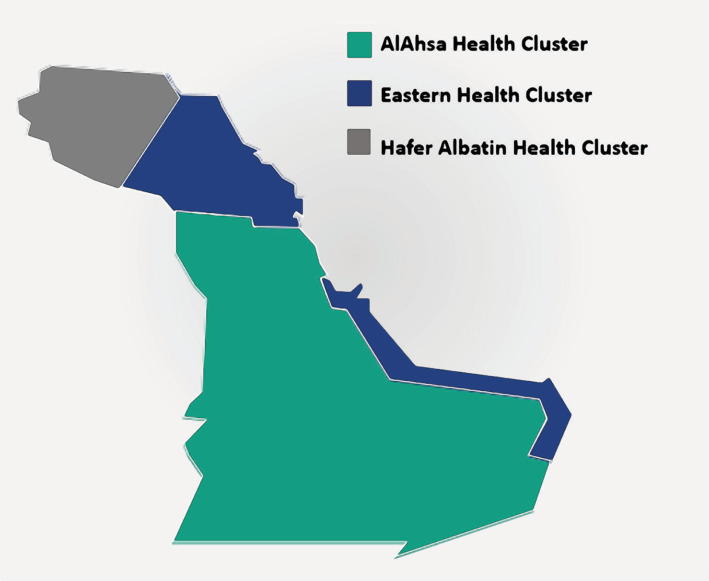
Eastern Region Clusters in Saudi Arabia.

During the transformation mandate, the Eastern Region was sectioned into three clusters: Eastern Health Cluster (EHC), AlAhsa Health Cluster (AHC), and HaferAlbatin Health Cluster (HBHC) (Figure 2).

In the Eastern region, the first cluster to pilot the MoC was the EHC in 2017 [12]. EHC was designated as one of the pathfinding clusters focusing on Chronic Care, specifically the interventions focused on case coordination, health coaching, and the prevention and management of type 2 diabetes mellitus [12]. EHC’s implementation of the MoC guided the development of the national pathway design manuals created by the VRO using the experiences and knowledge gained from the EHC experience.

The second Eastern cluster to implement the MoC was the AHC, which piloted the MoC in 2018 with the Breast Tumour Pathway, a process that included screening and the establishment of a One Stop Clinic (OSC) for diagnosis and treatment schedules. The Breast Tumour Pathways initiative was a key pilot used in benchmarking the MoC OSCs in the Kingdom. This was reflected in satisfaction KPIs showing a satisfaction rate of over 95% for patients and staff. Throughout the MoC journey, AHC has been one of the most advanced clusters at determining the population's needs and utilizing the MoC interventions to support those needs. The Breast Tumour OSC, for example, was implemented as a response to a high wait time for breast tumor diagnosis and an increased rate of external referrals to other clusters. In addition, AHC implemented a High-Risk Antenatal Pathway that addresses the increasing demand for high-risk maternal patients to reduce the load on the Maternity and Children Hospital.

In 2018, the Minister of Health at that time, Dr. TawfiqAlRabiah, declared 2019 the year of hypertension improvement [27]. In response to this announcement, the MoC team at the VRO piloted the hypertension full-patient pathway as one of the Chronic Care Systems pathways across the Kingdom. The hypertension pathway covers the entire patient journey, from screening and prevention to treatment across all layers of care. In addition, it covers three Chronic Care interventions, namely Chronic Care Screening, Case Coordination, and Continuity of Care.

Finally, HaferAlBatin Health Cluster launched the MoC in 2019 as an un-clustered area. The Cluster began with the hypertension pathway in response to the ministerial priority. Based on their interest in developing this program further, the Regional Health Directorate took the responsibility of approving and delivering the MoC Rollout Plan and implementing the prioritized interventions until Cluster activation in 2021. One of the top successes of the HBHC was within the Planned Care System with the successful development and implementation of the Bed Management Pathway. The current status of the MoC initiatives in each cluster is described in Table 2.

**Table 2:** Eastern Region Health Clusters Summary in 2022.

Cluster	Catchment population	Number of patients enrolled	Number of facilities	Number of planned initiatives	Number of initiatives implemented
**Eastern Health Cluster**	1,600,000	365,105	162	33	32
**AlAhsa Health Cluster**	650,000	174,723	80	36	19
**HaferAlbatin Health Cluster**	467,000	215,449	47	20	20
**Total**	2,717,000	755,277	289	89	71

### MoC successes in the Eastern region

The implementation of the MoC in the Eastern region has yielded significantly positive results. As documented in internal reports, patients have reported positive encounters and improvements in their overall experience. The impact assessment conducted in March 2022 using primary data from Cluster Health Information Systems (HIS) and Press Ganey patient satisfaction surveys showcased the outcomes of the MoC and helped to estimate the projected opportunities by 2030. One example of a successful intervention was the Health Hotline, a program listed in Table 1 under Cross-Cutting Interventions. The Health Hotline allows patients to receive virtual care at the convenience of their homes, around the clock, seven days a week. Caregivers found support from healthcare providers who had direct contact with the palliative medicine consultant and access to the hospital information system, which contained their health records and medication information. The updated system enabled caregivers to provide tailored recommendations related to symptom management based on patients’ needs. When faced with uncontrolled symptoms, patients could avoid unnecessary emergency visits and inappropriate admissions to the hospital by receiving advice over the phone, thereby reducing the psychosocial and physical burdens of waiting in emergency departments. Additionally, this program resulted in cost savings for patients and the Kingdom by reducing the need for hospital services. The impact assessment found that 78% of palliative patients avoided an emergency department visit due to the specialist palliative care phone service in the Eastern Health Cluster.

Another key benefit of the MoC is its focus on prevention rather than just treating illnesses and chronic health conditions after they have already occurred [28]. For example, the Health Coach Program has transformed patients from being sick and hospitalized to becoming healthy and managing their health from home. Health coaches help patients develop clear, measurable smart health goals with clear timelines to help them improve their overall life [29]. This program also encourages patients to adopt a healthier lifestyle with better nutritional choices, active physical life, and psychological and social support [29]. The impact assessment showed that out of 495 patients who were enrolled in the smoking cessation program, part of Health Coach within EHC, in 2022, 24% (154) achieved their goals of totally quitting smoking within 6 months. The updated program also offers improved data management, collection, and visualization and better engagement with patients and the community.

## Discussion

### Challenges to the Model of Care implementation

The MoC was developed in response to the challenges and projections outlined in previous sections; however, as expected with any major reform, the implementation of the MoC faced several challenges.

The MoC began before other healthcare transformation programs and with no available budget. The lack of clear precedent and funding sources led to issues in integration and change management and inadequate optimization of resources. This finding aligns with Khalil et al. (2018), who reported that Saudi Arabia still has a long way to go in terms of change management and integration of health services [9]. The authors recommended developing policies and regulations to guide the integration of medicine in the transforming Saudi healthcare system.

Our study also reports the absence of a change management strategy. Although stakeholder engagement and communication plans were developed, no comprehensive awareness campaigns existed. The change management efforts were met with low levels of buy-in, and there was no detailed, customized strategy to meet the needs of the MoC. Similarly, Alharbi (2018) concluded that the focus in Saudi Arabia should be on involving the healthcare personnel responsible for implementing and maintaining the reform, with strong commitment from leadership in planning and policy formulation [30].

Another challenge was insufficient locally advanced clinical expertise and project management capabilities within teams and across regions. Some regions within the Kingdom lack specialized clinical expertise to guide the MoC pathway design locally, and most MoC teams had clinical backgrounds with limited knowledge and experience in project management, quality, and change management. The MoC also lacked some required enablers necessary for a successful implementation. For instance, E-Health initiatives were expected to be mandated nationally. Therefore, the clusters did not have the freedom to choose their preferred health information system and acquire their individual digital solutions. A study by Zaman et al. (2018) identified different obstacles to e-health implementation, which included a lack of staff training and staff resistance to using the electronic system [31].

Another critical challenge faced during the MoC implementation was the constantly changing requirements. The VRO requirements were based on current priorities instead of being set before the implementation to measure project completion and patient outcomes. Subsequently, it was not possible to measure outcomes and project success without clear requirements or success criteria. Additionally, there were no clear KPIs and requirements for quality and data management on a national level, leading to inconsistent data collection. Finally, due to an unclear current state, the unavailability of baseline data, and no clear current process, it was challenging to measure improvements and prioritize interventions within the MoC.

There are a number of suggestions to respond to the challenges mentioned above to support the MoC implementation in the Eastern region. Firstly, it is essential to develop and communicate a clear change management strategy at a national level and tailor it to the specific needs of each region. Secondly, there is a need for full-time employees dedicated to MoC projects to ensure that expected outcomes are met. Currently, most MoC employees are assigned part-time, which can lead to delays in data collection, communication, availability, follow-up, delivery, and high turnover rates. Therefore, we recommend dedicating full-time employees to maximize the Model of Care potential. Thirdly, integrated HIS systems between all clusters, but especially between hospitals and primary care facilities within a cluster, are recommended to support continuity of care. This would enable better care coordination, referrals, and case management. Lastly, to gain insight into the current state, we encourage the development of national and regional patient and disease registries that will aid in the overall understanding of the needs and trends of the catchment population of the cluster.

### Lessons learned

Capturing lessons learned from healthcare transformation should be an ongoing effort, crucial for improving current and future projects. Some of the lessons learned from the Eastern region while implementing the MoC were:

- The inconsistent pathway development process at regional and national levels inspired the development of ICCPs national standard pathway designs to support the consistency of care and provide quality measures throughout the clinical pathways;
- A lack of knowledge management between different regions and within clusters was recognized. This encouraged a culture of documentation and work handover. In addition, knowledge ambassadors were trained and certified to be responsible for knowledge management activities within their cluster, including capturing lessons learned and identifying knowledge-sharing needs;
- The low buy-in and resistance to change were evident during the MoC implementation. Change management is integral to any transformation, especially in a healthcare setting. To engage stakeholders, the Dream Team project was established to enhance the culture of change management within clusters. In addition, a Train the Trainer initiative was established to train employees to better support change management within their clusters. Change ambassadors were also appointed to facilitate and support changing the culture and mindset of the healthcare providers in regard to the transformation within their facilities through sessions, activities, and workshops to engage the staff;
- To help the MoC staff complete all planned MoC projects and stay on track with the suggested scope, pathways that reached maturity level were handed over to business as usual, and the clinical pathway management was moved to the operational counterparts within the cluster. This provided the MoC team with opportunities to design new innovative pathways based on cluster needs, with the maturity of pathways measured based on success criteria for each respective pathway;
- The early head start of the MoC prior to other transformational activities resulted in a lack of direction and clarity for the teams. Hence, the Implementation Framework was developed retroactively to guide the development of MoC projects in a way that considers cluster/region-specific needs and uniqueness;
- The inability to benchmark on a national level prompted the introduction of national KPIs developed to create a more accurate way to assess patient outcomes and pathway success between and within regions.

## Conclusion

The Kingdom of Saudi Arabia has been going through a remarkable transformation since the launch of Vision 2030 in 2016. The major achievements in all sectors testify to Saudi Arabia’s commitment to achieving Vision 2030 goals to build a better future for the country. The healthcare sector is no exception. Implementing the new MoC has provided a clear direction for the healthcare sector in Saudi Arabia and has resulted in improved data management, collection, and visualization and better patient and community engagement. Compared to the previous treatment-focused model, the MoC has also shifted the focus toward a prevention model.

The achievements in the Eastern region and the challenges and lessons learned discussed in this paper provide valuable insights into what has been accomplished so far. This paper also describes recommendations and enhancements in the processes and procedures to better achieve the health transformation goals. However, the literature on the Saudi health sector transformation and the MoC is currently scarce. Therefore, future studies are needed to measure the success of MoC pathways and their impact on healthcare provision and overall population health. Furthermore, there is a need for studies that showcase the impact of the MoC nationally and the uniqueness of the MoC experience in different regions in Saudi Arabia.
